# Genome Analysis of *Streptococcus pyogenes* Associated with Pharyngitis and Skin Infections

**DOI:** 10.1371/journal.pone.0168177

**Published:** 2016-12-15

**Authors:** Joe Ibrahim, Jonathan A. Eisen, Guillaume Jospin, David A. Coil, Georges Khazen, Sima Tokajian

**Affiliations:** 1 Department of Natural Sciences, Lebanese American University, School of Arts and Sciences, Byblos, Lebanon; 2 University of California Davis Genome Center, Davis, California, United States of America; 3 Department of Computer Science and Mathematics, Lebanese American University, School of Arts and Sciences, Byblos, Lebanon; Tianjin University, CHINA

## Abstract

*Streptococcus pyogenes* is a very important human pathogen, commonly associated with skin or throat infections but can also cause life-threatening situations including sepsis, streptococcal toxic shock syndrome, and necrotizing fasciitis. Various studies involving typing and molecular characterization of *S*. *pyogenes* have been published to date; however next-generation sequencing (NGS) studies provide a comprehensive collection of an organism’s genetic variation. In this study, the genomes of nine *S*. *pyogenes* isolates associated with pharyngitis and skin infection were sequenced and studied for the presence of virulence genes, resistance elements, prophages, genomic recombination, and other genomic features. Additionally, a comparative phylogenetic analysis of the isolates with global clones highlighted their possible evolutionary lineage and their site of infection. The genomes were found to also house a multitude of features including gene regulation systems, virulence factors and antimicrobial resistance mechanisms.

## Introduction

*Streptococcus pyogenes*, also referred to as Group A Streptococcus (GAS) for harboring Lancefield group A antigen, is a clinically important human pathogen [1]. Despite its limited prevalence in modern times as compared to other pathogens, the myriad of infections it causes are still commonly lethal [2]. Streptococcal infections range from localized throat infections such as tonsillitis or pharyngitis, to invasive infections such as sepsis, necrotizing fasciitis and streptococcal toxic shock syndrome (STSS) [1, 2]. Most of the infections are seen in children aged four to seven years [3], with penicillin still being effectively used for treatment. Drug resistant clones however, have been increasingly reported globally [4, 5], and were attributed to environmental and intracellular resistance mechanisms [6]. Treatment complications usually arise with the use of other antimicrobial agents when the patient is allergic to penicillin.

A number of genome-encoded virulence factors such as pili, M proteins, leukocidins, streptolysins, complement inhibiting proteins, immunoglobulin-degrading enzymes, and superantigens have been detected in *S*. *pyogenes*, [1, 2], in addition to efflux pumps and leukocyte evasion strategies [7]. Interestingly horizontal gene transfer (HGT) and prophage integration are common amongst *S*. *pyogenes* genomes giving them plasticity and genomic variation [7]. These factors collectively confer additional virulence and resistance capabilities and alter the regulation of existing genes [8]. The *emm* gene, encoding the M protein contains conserved, semi-conserved, and hypervariable regions, and is thus used as an epidemiological marker for GAS [9]. *emm* occurrence amongst GAS strains is linked to geographic localization [10].

Globally, the GAS disease burden is not yet fully quantified, 2005 estimates showed that a minimum of 18.1 million people were suffering from invasive diseases, with another 111 million cases of streptococcal pyoderma and 616 million cases of pharyngitis recorded. 1.78 million additional incident cases were estimated to occur each year [11]. In the Middle East and in Lebanon in particular, studies linked to the emergence and epidemiology of *S*. *pyogenes* are still very limited [11]. Previous studies, focused at establishing *emm* and pulse field gel electrophoresis (PFGE) based typing schemes [12, 13]. *emm*1, 22, and 28, which were the most common types, were reported as being susceptible to penicillin and vancomycin but resistant to erythromycin and clindamycin [12]. Karaky et al. in a more recent study detected 33 *emm* types and subtypes with the dominant being *emm*1, *emm*22, *emm*28, *emm*88 and *emm*4 and with 10% of the isolates being resistant to erythromycin and 3% resistant to erythromycin and clindamycin [13]. In this study we sequenced the genomes of nine *S*. *pyogenes* isolates representing the most commonly recovered *emm* types to build on the previous findings and to elucidate the molecular epidemiology, genomics, and phylogenomics of this important human pathogen.

## Materials and Methods

### Ethical Approval

Ethical approval was not required as clinical isolates were collected and stored as part of routine clinical care. Clinical isolates and patient records/information were anonymous and de-identified prior to analysis

### Bacterial isolates and genomic DNA extraction

This study was conducted on nine *S*. *pyogenes* bacterial isolates previously collected from the American University of Beirut Medical Center (AUB-MC). The samples were recovered from throat and pus swabs of patients with streptococcal infections during the period from August 2010 to November 2011, and were chosen to cover the common *emm* types in the country (Table 1). The isolates were cultured overnight on Trypticase Soy Agar (TSA) (Bio-Rad, USA) medium. DNA was extracted using the Nucleospin Tissue kit (Macherey-Nagel, Germany) following the manufacturer’s instructions.

**Table 1:** Epidemiological Information on clinical *S*. *pyogenes* isolates. The relationship between *emm* types [14], MLST types, and diseases [15] in addition to site of isolation. emm pattern A-C is usually linked to upper respiratory tract infections (URT), pattern D is linked to skin infections while pattern E represents a generalist group.

**Sample Name**	**Sex**	**Age**	**Origin**	**Specimen**	**Disease**	***ST*-Type**	***emm* typing in [12]**	**Virulence Factors in [13]**	***emm* Pattern**	**Tissue preference/Associated Disease**
**SP1**	F	48	Lebanon	Swab, throat	Pharyngitis	ST-36	12	Cys. Prot. B—SpeL	A-C	Throat/URT
**SP2**	F	47	Lebanon	Swab, pus	Dermatitis	ST-304	108	Cys. Prot. B—SpeL	D	Skin/-
**SP3**	M	7	Lebanon	Swab, throat	Pharyngitis	ST-101	89	Cys. Prot. B—SpeL	E	No preference/URT
**SP4**	M	8	Lebanon	Swab, throat	Pharyngitis	ST-52	28	Cys. Prot. B—SpeL—ssa	E	No preference/ Invasive
**SP5**	M	35	Lebanon	Swab, pus	Dermatitis	ST-28	1	SpeA—Cys. Prot. B—SpeL	A-C	Throat/Invasive
**SP6**	F	23	Lebanon	Swab, throat	Pharyngitis	ST-101	89	SpeG	E	No preference/URT
**SP7**	M	4	Lebanon	Swab, throat	Pharyngitis	ST-46	22	SpeA—Cys. Prot. B—ssa—smeZ	E	No preference/Invasive
**SP8**	F	1	Lebanon	Swab, throat	Pharyngitis	ST-109	85	SpeA—SpeH—ssa	D	Skin/Skin
**SP10**	M	7	Lebanon	Swab, throat	Pharyngitis	ST-167	118	Cys. Prot. B—SpeH—SpeK—smeZ	E	No preference/-

### Genome sequencing

DNA extracted from each *S*. *pyogenes* isolate (50-ng/sample) was prepared for sequencing with the use of the Nextera XT DNA Sample Prep Kit (Illumina). Clean up was performed using the AMPure XP PCR purification beads (Agencourt, Brea, CA, USA). The resulting individual DNA libraries with fragment sizes ranging from 500–1000 bp were quantified by quantitative PCR on a CFX96 (Bio-Rad, USA) in triplicate at two concentrations, 1:1000 and 1:2000, using the Kapa library quantification kit (Kapa Biosystems, Woburn, MA, USA). Based on the individual library concentrations, equimolar pools of the indexed libraries were prepared at a concentration of at least 1 nM using 10 mM Tris-HCl (pH 8.0) and 0.05% Tween 20. Pooled paired-end libraries were sequenced to a read length of at least 250 bp.

### Genome assembly

*De novo* assembly of the sequenced genomes was done using A5 assembler (v. 20130627) with default assembly parameters. This pipeline automates the processes of data cleaning, error correction, contig assembly, scaffolding, and quality control [16, 17].

### Genome annotation and gene detection

The assembled genomes were annotated using the RAST server (http://rast.nmpdr.org) that uses subsystems technology to assign gene function. RAST was also used for the identification of protein encoding genes, rRNAs, and tRNAs [18, 19]. The SEED viewer service from RAST in combination with the web tools provided by the Center for Genomic Epidemiology (CGE) website (www.genomicepidemiology.org) were used to generate a detailed list of the genetic elements in question. The ResFinder 2.1 web server was used to identify acquired antimicrobial resistance genes present on the bacterial genome [20]. Due to ResFinder’s inability to confirm the functional integrity, and levels of gene expression and resistance arising to acquired mutations in housekeeping genes, a hybrid resistance profile was generated using the ResFinder hits in addition to phenotypical data from previously published studies. The VirulenceFinder 1.2 service from the same website was used to identify additional virulence-specific genes [21]. The PathogenFinder 1.1 service from CGE, was used to obtain an overview of the genomic pathogenic gene families [22].

### Multi-locus sequence typing (MLST)

MLST typing of the isolates was carried out using CGE’s MLST 1.7 server to detect sequence polymorphisms within the *gki*, *gtr*, *muri*, *muts*, *recp*, *xpt*, and *yqil* genes [23].

### Phage and mobile element detection

Phage detection was done using the publicly available Phage Search Tool (PHAST) (http://phast.wishartlab.com/index.html) [24]. This tool provides the closest identity match for detected phages in addition to their site of integration. Putative insertion elements were double checked using BLASTx with an identity threshold of 80%. Putative phage insertion sequences were then annotated using RAST in order to determine the genes they encode.

### Phylogenetic analysis

To determine the phylogenetic relatedness a concatenated marker gene maximum-likelihood tree was constructed using a number of *S*. *pyogenes* reference genomes chosen based on BLAST similarity results, clonal complexes and sequence types (MGAS6180 CP000056, MGAS10394 CP000003, M1476 AP012491, M1GAS SF370 NC002737, NZ131 CP000829, A20 CP003901, 7F7 PRJNA238516, and *S*. *pneumoniae* R6 AE007317 as an outlier strain). The genomes were first processed with PhyloSift [25], the tree was then constructed using FastTree [26], visualized and edited with Dendroscope [27]. Pairwise alignment and visualization of our selected genomes with the respective reference strains was achieved through the Mauve aligner [28] using defaults settings.

### Nucleotide Sequence Accession Numbers

The whole-genome shotgun projects have been deposited at DDBJ/EMBL/GenBank with accession numbers AYPA00000000 (SP1-LAU), AWOZ00000000 (SP2-LAU), AWPA00000000 (SP3-LAU), AWPB00000000 (SP4-LAU), AWPC00000000 (SP5-LAU), AWPD00000000 (SP6-LAU), AWPE00000000 (SP7-LAU), AWPF00000000 (SP8-LAU), and AWPG00000000 (SP10-LAU) [29].

## Results and Discussion

Sequencing resulted in an average of 2,503,465 paired-end reads per isolate, with the average being 1,976,732 high-quality reads following quality trimming and error correction. The average sequence coverage of the whole genomes was 293X and the minimum sequence coverage was 85X for the SP6 isolate. The average N50 for the assemblies was 196,439 bp with the lowest being 98,990 bp for SP7. The initial assemblies resulted in an average of 77 contigs per isolate all of which greater or equal to 500 bp in length. During scaffolding, some contigs were merged based on short overlaps and read-pair information, yielding a reduced final average of 70 contigs per isolate. The complete details and statistics of the sequencing and assembly results for each isolate are shown in Tables 2 & 3 [29].

**Table 2:** Genome assembly statistics.

	SP1	SP2	SP3	SP4	SP5	SP6	SP7	SP8	SP10
**Genome size (bp)**	1,925,871	1,727,943	1,745,842	1,906,369	1,813,544	1,733,546	1,953,601	1,917,411	1,771,196
**Number of contigs (> = 500 bp)**	155	29	21	28	26	19	158	186	12
**Average contig size (bp)**	12,425	59,584	83,135	68,085	69,752	91,239	12,365	10,309	147,600
**Longest contig size (bp)**	343,437	653,370	659,470	249,942	752,626	674,975	207,328	250,346	781,011
**GC content (mol %)**	38.4	38.4	38.4	38.2	38.4	38.4	38.4	38.5	38.4
**N50 (bp)**	209,002	280,940	167,926	205,587	308,655	116,374	98,990	104,600	275,797

**Table 3:** Gene prediction and annotation summary of the nine *S*. *pyogenes* isolates.

	SP1	SP2	SP3	SP4	SP5	SP6	SP7	SP8	SP10
**# of predicted genes**	1991	1694	1725	1905	1832	1698	2041	1971	1740
**# of pseudogenes**	22	26	27	24	18	30	31	37	34
**# of predicted potein-coding genes**	1907	1669	1711	1923	1824	1692	1937	1866	1740
**# of tRNA genes**	56	65	56	54	57	54	56	56	56
**# of rRNA genes**	7	7	6	6	9	4	5	10	4
**# of subsystems**	317	313	311	317	319	314	316	322	317

**Fig 1:**
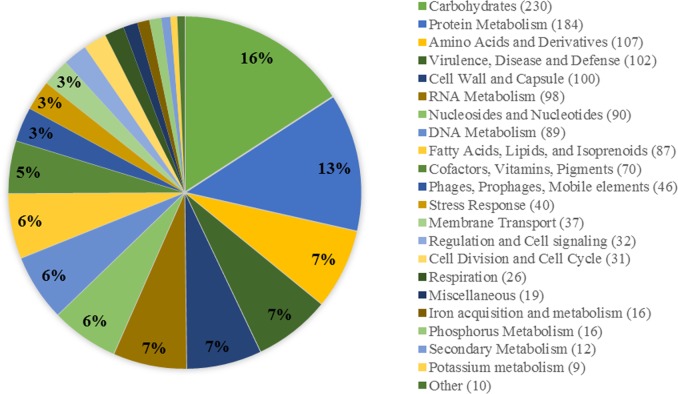
Average subsystem category distribution in the streptococcal genome. The pie chart shows the subsystem related genes as a percentage of the whole genomic content. Numbers next to the label entries indicate the number of predicted genes involved in a particular subsystem.

The average genome size was 1.83 Mbp and the G+C content ranged between 38.2% and 38.5% (Table 2) with an average of 38% both falling within the general ranges for the species [7]. An average of 1844 open reading frames (ORFs) was detected in the sequenced genomes, 1752 of which encoding proteins, 57 tRNAs and 6 rRNAs. The average gene size was 868 bp with coding sequences covering around 85% of the genomes (Table 3). This was consistent with the averages obtained within the used reference strains, and in general amongst *S*. *pyogenes* strains [30]. An average of 315 discrete biological subsystems were identified (Table 3) the majority of which were related to nutrient metabolism, virulence factor and bacterial cell wall synthesis subsystems (Fig 1). Only nine amino acid synthesis subsystems were detected, reflecting the fastidious growth requirements of *S*. *pyogenes* [31]. The inherent lack of biosynthetic pathways however, is offset by the relative abundance of membrane transport systems (37 subsystems)—that scavenge resources from the environment—including around 10 putative ABC transporters, used specifically for peptide uptake. Virulence factors and defense mechanisms make up on average 10% of the genome, with an equal percentage allocated to cell wall and capsule proteins. Considering the small size of the streptococcal genome, virulence related genes take up a significant part, which goes in line with *S*. *pyogenes* being a strict human pathogen [1]. The various sequence types (ST) identified based on MLST database search were: ST-36, ST-304, ST-101, ST-52, ST-28, ST-46, ST-109, and ST-167. ST were then correlated to *emm* types generated in an earlier study [12] as well as to the disease associated with these typing patterns (Table 2) and were found to be mostly compatible with the site of isolation.

### Gene Regulation

Sigma factors (σ factors), used for bacterial transcription initiation, can be highly variable. *Bacillus* spp. have up to 18 σ factors while *Haemophilus influenzae* has only three [32]. A total of three to four factors were detected in the studied isolates conforming to the number of σ factors found in small bacterial genomes (1–4 σ factors) [33]. σ^70^ (*ropD*) factor, the main σ factor of the species, and ComX were detected in all the isolates in this study. ComX, a homologue of the σ factor present in *S*. *pneumoniae*, transcriptionally regulates competence specific genes serving in DNA uptake and integration [34], and is encoded by two ORFs [35]. Sequence of an additional putative σ factor was detected in three of the isolates (SP2, SP3 and SP7), which could be linked to a minor heat competence transcriptional regulator found in *Escherichia* coli, σ^24^ [36]. The acquisition of σ^24^ might be an adaptation to help it survive, as *S*. *pyogenes* often encounters high temperatures inside the human host due to inflammatory responses and immune defenses [31].

Comparable to other microorganism, *S*. *pyogenes* responds to environmental changes by choosing the appropriate transcriptional signals [33] in addition to a variety of stress response proteins, specifically proteases and highly conserved stress regulation genes [31]. Osmoregulation is maintained by a two-component system comprising aquaporin Z and two osmotically inducible outer membrane proteins, OmpA and OsmY, the genes of which were detected in all the isolates. Choline and betaine uptake and biosynthesis genes (*opuAA*, *opuAB*, *opuAC*, *proV*, *proX* and *chA*) were also identified. These are highly conserved elements encoding proteins that can act as both osmoprotectants and energy sources [37]. Similar to other lactic acid producing streptococcal species, acid stress response in *S*. *pyogenes*, is achieved by proton translocation through the F_0_F_1_ ATP synthase [38]. Eight genes encoding structural parts of the ATP synthase as well as cold and heat shock response proteins were identified. CspA (*cspA)*, a cold shock response protein belonging to the Csp family of proteins, and which allows *S*. *pyogenes* to cope with temperature reductions, was also detected [39]. The heat shock response family of proteins was detected as part of the *dnaK* gene cluster that houses a range of protein families including ribosomal methyltransferases, and chaperones (DnaJ, DnaK). The *dnaK* gene cluster is still poorly characterized in streptococci, with the exception of its high conservation among firmicutes [40]. The main role of *dnaK* gene cluster is to prevent the aggregation of heat denatured proteins inside the cell following heat shock [41]. Its activation has been reported inside macrophages, during an infection with *Salmonella enterica* [41], which consequently could be related to pathogenesis.

Gene regulation in *S*. *pyogenes* is stringently controlled mainly in response to environmental changes, and often linked to the type of infection superficial versus invasive [42]. To date, studies have identified around 13 two-component regulatory systems in *S*. *pyogenes*, in addition to more than a hundred putative independent transcriptional regulators [43, 2]. Perhaps one of the most important regulons detected in all the sequenced genomes is the control of virulence regulatory system (covRS), otherwise known as the capsule synthesis regulon (csrRS). CovRS is a two-component system made up of a membrane-bound sensor kinase (CovS), and a DNA binding response regulator (CovR) [44]. The regulon governs streptococcal virulence in response to environmental conditions such as pH, temperature, and ion concentrations [2]. This controls the expression of 10 to 15% of the streptococcal genes either directly or indirectly [45]. CovRS positively regulates the expression of several virulence factors including the streptococcal cysteine protease SpeB, and the hyaluronic acid capsule synthesis proteins [42]. Unidirectional mutations in the covRS regulon have been reported to alter the expression of virulence factors affecting the progression of streptococcal invasive diseases [46]. The multiple-gene regulator (Mga) controls the expression of many virulence genes, including the M family of proteins, in response to environmental carbohydrate availability [47]. Mga regulation represents phase and metabolite-dependent regulation in *S*. *pyogenes*, and the regulon is essential for the organism when shifting from the colonization to deep tissue invasion [48].

Chromosomal replication is another important aspect of the microbial cell cycle; it is initiated by the binding of the DnaA protein on specific DnaA boxes located in the replication origin [oriC] [49]. With the increased availability in complete genome sequences, the proper identification of replication origins [oriCs] and their characterization has become essential for the analysis of bacterial whole genomes [50]. For our purpose, we used the web-based Ori-Finder system as described by Gao & Zhang [50] to identify oriCs and DNA boxes in our assembled genomes and the results subsequently BLASTed against the DoriC database to confirm the reliability of the prediction [51]. All of the isolates with the exception of SP7 and SP8, exhibited a 154 nucleotide oriC sequence similar to that found in the M1GAS SF370 strain. The average oriC AT content was 0.7078 and it housed 3 DnaA boxes; TGTGGAAAA, TTATCCACA and TTATCCACT. The OriCs of SP2 and SP4 were very similar to that of the MGAS6180 and A20 strain respectively. The SP8 oriC was found to be highly similar to that of the NZ131 strain being 232 nucleotides in length and having a 0.6897 AT content. Interestingly, a 173 nucleotide long oriC was identified in SP7 which was 63.01% composed of AT residues and interspersed by five DnaA boxes; three TTATCCACAs and two TGTGAATAA, a result that was uncommon amongst *S*. *pyogenes* as the species mostly possesses either three or seven DnaA boxes in the replication origin region an example of the latter being strains MGAS15252 and MGAS1882 [51]. Originally, DnaA boxes were shown to control microbial initiation mass [52], whereas more recent studies have outlined the organizational importance of the oriC region in influencing bacterial proliferation and by that invasion and pathogenesis [53]. Some researchers believe that this organization is conserved among bacterial species while others do not agree [54]. Our findings were in agreement with the former claim as the oriCs we detected in our biotypes were very similar to those found in the reference strains, all of which generally exhibited species-wide homology.

### Virulence Factors

*S*. *pyogenes* possesses an arsenal of virulence factors that targets and impairs the immune system [2]. Many of the genes constituting the streptococcal virulome were identified in the sequenced genomes (Table 4). Most of the virulence factors detected in this study, were scattered throughout the genome and were are not strictly part of a pathogenicity island, which was in harmony with previous findings [31]. For the most part, these elements are conserved amongst strains, apart from the ones carried by phages and prophages [55]. Genes coding for some exotoxins, including the streptokinase, hyaluronate lyase, and nicotine adenine dinucleotide glycohydrolase (NADGH), were highly conserved amongst the isolates (Table 4); these normally induce apoptosis in neutrophils and macrophages [2]. Interestingly, the cyclic AMP (cAMP) factor gene *cfa* was detected in all the sequenced genomes and reference strains. *cfa* was originally thought to be exclusive to Group B Streptococci (GBS) [56], however its presence was confirmed in GAS following the original sequencing work of Ferretti et al. [31]. The cAMP factor is an extracellular protein that causes synergistic lysis of host erythrocytes [57], with its role in pathogenesis not being fully understood. Numerous related streptococcal species are naturally competent for transformation via a pathway yet to be described [31], hence the presence of the *cfa* gene could be the outcome of mobilization and horizontal gene transfer (HGT).

**Table 4:** List of genes attributable to virulence traits in the streptococcal genomes.

**Virulence Trait**	**SP1**	**SP2**	**SP3**	**SP4**	**SP5**	**SP6**	**SP7**	**SP8**	**SP10**	**Gene(s) with potential for conferring virulence traits**
Antiphagocytic M protein	+	+	+	+	+	+	+	+	+	*emm*, *enn*X, *fbp*, *iga*R
Streptokinase	+	+	+	+	+	+	+	+	+	ska
CAMP factor	+	+	+	+	+	+	+	+	+	*cfa*
Streptolysin O	+	+	+	+	+	+	+	+	+	*slo*
Streptolysin S	+	+	+	+	+	+	+	+	+	*sag*B, C, D, E, F, H, I, asn-ORF, ABC transporter
Hyaluronate lyase precursor	+	+	+	+	+	+	+	+	+	*hyl*
Hyaluronan synthase	+	+	-	+	+	-	-	+	+	*has*A
Streptococcal pyrogenic exotoxin A*	-	-	-	-	+	-	-	-	-	*spe*A
Cysteine Protease B*	+	+	+	+	+	+	+	+	+	*spe*B
Streptococcal pyrogenic exotoxin C*	-	-	-	-	-	-	+	-	-	*spe*C
Streptococcal pyrogenic exotoxin G	+	+	+	+	+	+	+	+	+	*spe*G
Streptococcal pyrogenic exotoxin H*	-	-	-	-	-	-	-	-	-	*spe*H
Streptococcal pyrogenic exotoxin I*	+	-	-	-	-	-	-	+	-	*spe*I
Streptococcal pyrogenic exotoxin J	-	+	-	-	+	-	-	-	-	*spe*J
Streptococcal pyrogenic exotoxin K*	+	-	+	+	-	+	+	-	+	*spe*K
Streptococcal pyrogenic exotoxin L*	-	-	-	+	-	-	-	-	-	*spe*L
Streptococcal pyrogenic exotoxin M*	-	-	-	-	-	-	-	-	-	*spe*M
Streptococcal mitogenic exotoxin Z	+	+	+	+	+	+	+	+	+	*sme*Z
Streptococcal superantigen A*	+	-	-	-	-	-	+	+	-	*ssa*A
C5a peptidase	+	+	+	+	+	+	+	+	+	*scp*A
Secreted endo-beta-N-acetylglucosaminidase	+	+	+	+	+	+	+	+	+	*ndo*S
Streptococcal inhibitor of complement	-	-	-	-	+	-	-	-	-	*sic*
Exotoxin nucleases	+	+	+	+	+	+	+	+	+	*spd*1, 2, 3, 4, *sda*
Immunoglobulin-binding protease	+	+	+	+	+	+	+	+	+	*ide*S
Adhesins and invasins	7	4	6	7	3	6	9	7	6	*fba*, fibronectin binding proteins, serum opacity factors
Collagen-like surface proteins	+	+	+	+	+	+	+	+	+	*scl*A, B

. + and–indicate the presence or absence of genes respectively, numbers indicate the number of pertaining genes detected, while * indicates phage-encoded superantigens.

*Slo* and *sls*, encoding streptolysin S and streptolysin O, were also detected in all of the isolates (Table 4). SLO and SLS are leukocidins that disrupt host cell membranes and induce apoptosis in phagocytes [58]. SLS is virtually secreted by all GAS [59]; it increases streptococcal resistance to phagocytosis by distorting neutrophil membranes [60], in addition to disrupting erythrocytes, lymphocytes, and even platelets [61]. Individual *S*. *pyogenes* genomes can greatly differ in their genetic composition, mainly due to the acquisition of exogenous genetic elements either through HGT, or bacteriophage integration [62]. The *sic* gene, encoding the streptococcal inhibitor of complement and uniquely detected in M1 and M57 GAS strains [63], was detected in SP5 (*emm*1) (Table 4) and in the M1 GAS reference strain. This conforms our findings to the work done by Bahnan et al. [12]. Host neutralizing antibodies exert selective pressure usually leading to high polymorphism in the *sic* gene even within the same *emm* type [64], however this was not observed in our study with both the M1 GAS reference and SP5 having homologous *sic* gene sequences.

The endo-beta-N-acetylglucosaminidase of streptococci, EndoS (*ndo*S), is a secreted immunoglobulin degrading enzyme that enhances the organism’s ability to resist opsonophagocytosis, and is encoded by the 3 Kbp *ndo*S gene [2]. *ndo*S was detected in all biotypes (Table 4) with its size ranging from 2943 bp to 3015bp, these size differences can be attributed to possible insertions or deletions. Individual BLASTing of the sequences, revealed even distribution between the two protein isoforms EndoS and EndoS F2, which are similar enzymes but with different oligosaccharide specificity [65]. Both however, are distinct from the EndoS2 that was previously only detected in M49 GAS strains [66], another fact in agreement with the findings of Bahnan et al. [12]. The heterologous expression of the EndoS in GAS strains other than the M1 serotype however, should not be easily dismissed as it has been shown to enhance virulence in murine models of invasive streptococcal infections [67]. Genes encoding the streptodornase B (*spd*1), an exotoxin nuclease, were detected in all of the isolates as well as in the chosen reference strains (Table 4); conserved in size and in genomic location. The gene was part of an extended SpeB-SpeF regulon and is flanked by a transcriptional regulator and a low temperature response protein. These gene sequences shared homology with their counterparts on bacteriophages, specifically the *S*. *pyogenes* phages 315.3, 315.6, and phage 9. Despite being traditionally phage encoded [68], the uniformity of the location of these sequences in the genomes indicates that they could be fixed remnants of phage integration events over time. Streptodornase D, also encoded on phages 315.3 and 315.6, was detected in six of the studied isolates (Table 4), while absent in the chosen reference strains. However, SEED similarity matches linked it to MGAS5005 and MGAS9429 strains, further elucidating the possibility of HGT. The immunoglobulin-binding protease, IdeS (*ide*S), was detected in all the biotypes (Table 4). BLAST revealed the protein products to be of the Mac-1 isoform with none being related to Mac-2 [69]. Despite impairing neutrophil mediated phagocytosis [70], IdeS is not essential for phagocyte resistance [71], which could explain the lack of an evolutionary pressure to drive diversity among the products of this gene.

**Fig 2:**
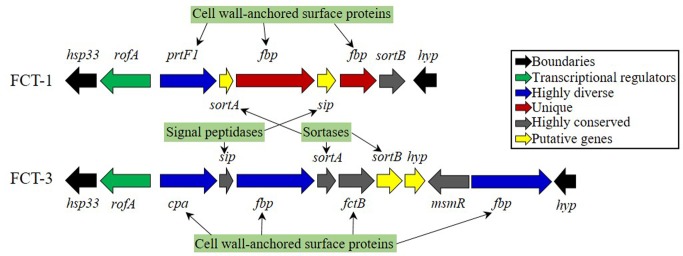
Fibronectin-Collagen-T-antigen (FCT) region organization of FCT types 1 and 3. FCT-1 (SP8) was identified in only one of the isolates whereas FCT-3 was seen in all of the rest. The region is flanked by a chaperon (*hsp*33) and a hypothetical protein (*hyp*). In between are mostly cell wall-anchored proteins namely fibronectin binding proteins (*fpb* namely *prt*F1/2) and collagen binding proteins (*cfa*). Sortases (*sort*) that modify surface proteins are also found in addition to signal peptidases (*sip*). A combination of unique, highly diverse, and highly conserved loci determines the FCT type.

Three to nine fibronectin binding (FBP) and collagen-like surface protein genes were additionally detected in the tested isolates (Table 4), these were present in three major genomic locations one gene was found downstream of the C5a peptidase gene, another set (two to three genes) was found downstream of the streptopain/streptopain inhibitor gene set, and the remaining genes were located in FCT region. These genes have different characteristics depending on *emm* type [72]. The FCT region, considered “a hot intergenomic recombinatorial site”, is a ≈11 to 16 Kbp chromosomal region flanked by highly conserved genes, a chaperon and a hypothetical protein, between which a unique combination of conserved and semi-conserved loci was present [73, 74]. The organization of this region was determined manually by examining the gene loci in the isolates and comparing it with similar loci on known references. To date, nine distinct FCT variants have been reported [74]. All the biotypes had the FCT-3 arrangement with the exception of SP8, which belonged to the FCT-1 (Fig 2). FCT-1 is usually rare amongst GAS isolates and can be can be detected in M1 and M6 strains such as the SF370 and MGAS10394. After further sequence annotation the SP8 FCT-1 configuration was determined to be similar to that present in the M6 serotype containing the distinctive T-antigen backbone protein and the ancillary protein FctX. The FCT-3 configuration was comparable to that present in M3 and M49 serotypes with the putative chaperon protein SipA [74]. Additional putative genes were detected as part of the locus, these were mainly encoding sortases and signal peptidases. Clinically, FBP contributes to the adhesion of *S*. *pyogenes* cells to host cells, and enhances resistance to phagocytosis by averting the C3 convertase from depositing on the bacterial cell [75]. These proteins are vital to the pathogenesis of *S*. *pyogenes*; mutations were associated with a decrease in the epithelial cell adhesion efficiency by up to 10% in murine models [76, 77]. Even in GAS strains where high-affinity plasminogen binding proteins are not expressed, but FBP is present instead, plasminogen-mediated virulence can still be activated through a stable cell-associated enzymatic activity that lyses fibrin clots [78]. *Prt*F1 was identified in all the genomes, while *cpa* in six of them; both gene products could prime skin and throat infections [74] as is the case with our isolates.

The bacterial C5a peptidase (*scp*A), was detected in all isolates with an approximate size of 3550 bp (Table 4), it was identified strictly as part of the Mga regulon [79]. The locus extending from the *sme*Z gene through the *mf1* gene to the Mga regulon, is one of the best studied loci in the streptococcal genome for its virulence factors and inherent regulatory components [31]. This regulon is an independent, ubiquitous, multiple-gene regulator that controls the expression of numerous virulence genes namely those encoding the M family of proteins (*emm*, *mrp*, *arp*, and *enn*) that contribute to adhesion, invasion, and host immune evasion [47, 80]. It also governs the transcription of other non-M protein genes (*si*c, *scp*A, *scl*A) [81]. Mga regulates the expression of about 10% of the streptococcal genome [80] (Hondorp and McIver, 2007). In this work, regions associated with the Mga regulon showed high variability in genetic content; these included multiple M protein genes in addition to the C5a peptidase. BLAST results of the individual gene sequences showed the presence of the *emm* gene in all biotypes complemented by a gene encoding a FBP in six of the isolates (SP2, SP3, SP6, SP7, SP8 and SP10) as well as in the MGAS6180 reference strain. An immunoglobulin A receptor (*iga*R) was also detected in six of the isolates (SP2, SP3, SP4, SP7, SP8 and SP10). SP6 additionally carried the *enn*X gene in the operon, which was homologous to the one present in the reference strain NZ131 (Table 4). The genetic diversity of the Mga regulon detected here is in accordance with previous findings [31, 72, 80]. This diversity correlates with both tissue tropism and disease manifestation [72, 80]; usually, strains presenting one M family protein are limited to throat infections, and those exhibiting three or more proteins are implicated in more invasive infections [82]. Six of our isolates (SP2, SP3, SP4, SP6, SP7 and SP10) had M family protein genes, demonstrating their potential in causing invasive infections.

Clinically, many consider superantigens to be more significant than *emm* types in disease manifestation [83]. These pyrogenic exotoxins are perhaps the most important of all the streptococcal antigens [84] due to their ability to over stimulate the human immune system and contribute to tissue inflammation [85]. Excluding SpeG, SpeJ, and SmeZ, all superantigens are phage encoded [86]. Particular serotypes, such as M types 1, 3, 5, 6, 14, 18, 19, and 24 are normally associated with throat infections and rheumatic fever, while other such as M types 2, 49, 57, 59, 60, and 61 are linked to pyoderma and which are associated with pyoderma and acute glomerulonephritis [87, 88]. In this study, seven distinct Sag profiles were identified, with SpeGKZ being the most prevalent (three out of nine) (Table 4). The conserved gene product SpeB, along with SpeG and SmeZ were detected in all the isolates as well as the used reference strains. SpeH on the other hand, has been reported to have low prevalence [89, 90] and was not detected in any of the isolates or reference strains. This superantigen, detected in M12 MGAS9429 and M1 GAS160490 strains for example, is considered a variable characteristic in different strains and is not attributed to distinct *emm* types [91]. Our findings were consistent with the worldwide dissemination of the species [90], and with previous findings [9]. The superantigen profile of SP1 which (SpeGIKZ) matched that of the M5 Manfredo strain that is associated with rheumatic fever [92]. The SP2 profile (SpeGJZ) can be associated to skin infections as it housed the same superantigens as the M59 MGAS15252 strain, a serotype isolated from pyodermal infections [93]. SP3, SP6, and SP10 (SpeGKZ) were similar in superantigens to the puerperal sepsis strain M28 MGAS6180 only lacking SpeJ [94]. SP4 was the only isolate to house SpeL (Table 4), this antigen was first detected in M3 serotype isolates causing toxic shock-like syndrome (TSLS) cases, and it transfer to the *S*. *pyogenes species* was attributed to phage HGT [95]. The SAg profiles of SP1, SP3, and SP6 also matched *emm* types 12 and 89 isolates, which are linked to puerperal sepsis and cellulitis [9]. Interestingly, SP5 of *emm* type 1 was the only isolate having SpeA, which also present in the M1 MGAS5005 and M1 GAS160490 reference strains. SpeA is a characteristic unique to *emm*1 types and associated with severe infections such as kidney failure [96, 97]. SP7’s profile matched that of M3 MGAS315, indicating the implication of these two strain’s in throat infections. SpeI, which has a very low prevalence [9], was found to be present in SP8 in addition to SpeG, Z and SSA. This contrasted previous findings that revealed the co-occurrence of SpeI and SpeH, on the same bacteriophage (φ370.2) [31], our findings were more harmonious with those of Commons et al. [86] who reported that SpeI may have been lost during integration in the streptococcal genome, or that it was encoded by a gene carried on an entirely different phage, both of which can explain the low prevalence of this SAg [90, 9]. SP8 was similar to the M4 MGAS10750 strain associated with pharyngitis and scarlet fever [98], with the addition of SpeI. SmeZ normally exhibits a mosaic structure and a wide allelic variation amongst *S*. *pyogenes* strains in an attempt to escape antibody neutralisation [89]. Three variant types were detected in our isolates, SP1 and SP8 were of the smez-3 type, SP3, 4, 6 and 7 were of the smez-8 type, while SP2, 5 and 10 were smez-12. These alleles can be correlated (but not limited) to single *emm* types, including *emm* 12, *emm* 59 and *emm* 89 respectively [89]. M59 MGAS15252 and M12 MGAS2096 strains were also found to have the corresponding SmeZ types 3 and 8 respectively. None of the variants were found to have the nonfunctional single base deletion, leaving SMEZ to propagate GAS pathogenesis.

### Antimicrobial Resistance

Penicillin is still the standard drug for the management of streptococcal infections due to the organisms’ lack of natural resistance against it, with macrolides being alternatively used in patients with penicillin hypersensitivity [99, 2]. This led to the development of resistant strains either through efflux pumps (*mef*A) or through the ribosomal methylases (*erm*) [100]. All of the isolates undertaken exhibited an ABC transporter membrane-spanning permease linked to macrolide resistance (Table 5). These results matched with the ones previously reported by Karaky et al. [13], and supported the increase in macrolide resistance among *S*. *pyogenes* strains [12,13]. In the Middle East, macrolide resistance is at 10%-23%, which is significantly higher than in developed countries such as Germany (2.6%), Belgium (3.3%), and Spain (7.6%), where a restrictive use of macrolides was instituted [101, 102, 103].

**Table 5:** List of gene products identified in the isolates conferring resistance to respective antimicrobial agents.

**Gene Product Features**	**SP1**	**SP2**	**SP3**	**SP4**	**SP5**	**SP6**	**SP7**	**SP8**	**SP10**	**Antimicrobial Resistance**
DNA gyrase subunit A	+	+	+	+	+	+	+	+	+	Fluoroquinolones
DNA gyrase subunit B	+	+	+	+	+	+	+	+	+	
Topoisomerase IV subunit B	+	+	+	+	+	+	+	+	+	
Topoisomerase IV subunit C	+	+	+	+	+	+	+	+	+	
ABC transporter membrane-spanning permease	+	+	+	+	+	+	+	+	+	Macrolides
Translation elongation factor G	-	-	-	-	-	-	+	+	-	Tetracyclines
Tetracycline resistance protein TetM	-	-	-	-	-	-	+	+	-	
Multidrug resistance efflux pump PmrA	+	+	+	+	+	+	+	+	+	MDR
Multi antimicrobial extrusion protein (Na(+)/drug antiporter)	-	-	-	-	-	-	+	+	-	

+ and–indicate the presence or absence of genes respectively.

Fluoroquinolone resistance is another feature of GAS that has been reported by several studies [104, 105, 106], it occurs at two levels: a low-level resistance due to mutations in the quinolone-resistance-determining region (QRDR) in the topoisomerase IV (*par*C and *parE*) [102], and a less-frequently prevalent high level [107] caused by additional mutations in *gyr*A and *gyr*B genes of DNA gyrase [106]. In this study both levels of fluoroquinolone resistance were detected (Table 5). Our findings are concordant with the global increase in fluoroquinolone resistance, with resistance rates over a two-year span in Belgium and Spain, rising from 4.3% and 1.9% to 21.6% and 30.8% respectively [106, 104]. In contrast, resistance in the studied isolates to fluoroquinolones was not limited to a set of *emm* types.

Tetracycline resistance in *S*. *pyogenes* is usually conferred through ribosomal protection genes namely *tet*M and *tet*O [108], and the elongation factors EF-G and EF-T [109]. SP7 and SP8 carried tetracycline resistance factors, specifically *tet*M and EF-G (Table 5), suggesting a multi-drug resistant European clone lineage [110]. The same isolates also exhibited a Multi antimicrobial extrusion protein (Na(+)/drug antiporter) efflux pump conferring tetracycline resistance. Although not very common amongst streptococci [111], this pump is possibly analogous to the one detected in *S*. *aureus* [112]. It is noteworthy in this respect that tetracycline resistance conferring genes are often associated with macrolide resistance genes on the same mobile element, hence the unregulated use of tetracycline and macrolides may reciprocally augment resistance to both agents [113, 114].

### Bacteriophages, mobile elements and genomic recombination

In Gram-positive organisms, HGT transduction via bacteriophages often causes the most important genomic alterations and confers pathogenic traits [115]. The streptococcal genome in particular is highly dynamic owning to the numerous phage integration sites and transposable elements that can make up to 7–14% of the total genome [31, 116]. Phage-encoded genes are largely responsible for the pathogenesis and invasiveness of *S*. *pyogenes* [2]. Moreover, phages widely contribute to the diversity of *S*. *pyogenes* strains; approximately 90% of the streptococcal genomic content is shared even among different serotypes with the exclusion of unique prophage-bound sequences [117]. Numerous confirmed and putative prophages and prophage-like elements were detected (Table 6). The ф315.x family of *S*. *pyogenes* specific phages was the most prevalent type followed by the P9 bacteriophage. These phage loci were found encoding streptodornases A and B and a hyaluronidase, which are major virulence factors. It is noteworthy that the same set of prophages were integrated at different positions within the tested isolates, indicating genomic rearrangements [72]. The ф315.x phages encoded phage holin genes, these are associated with hot phage recombinatorial regions further adding to the variety introduced by phage integration events [72]. Interestingly, the same phage sequences (ф 315.2) were detected three times in the SP1 genome (Table 6). This is attributed to the fact that phage integration is an ongoing process, with some of phage sequences being probably remnants of old integration events [94], and/or the outcome of new acquisitions. Additionally, similar phages can code for different gene sets and thus still contribute to streptococcal genomic diversity even with multiple infections of the same phage. Non species-specific phages were also identified in our isolates, namely *Bacillus spp*. and *Enterococcus spp*. phages, these stress again at phage DNA uptake being an important route through which *S*. *pyogenes* acquires genetic determinants to propagate and thrive in the host. Given that the species is a strict human pathogen, the actual mechanisms through which phages were acquired are not well understood. Previous work attributed this phenomenon to the presence of small signaling molecules in phages, analogous to those present in mammalian cells. Such molecules act as inducers, activating phage induction only when the environmental conditions are optimal to accommodate a competent recipient host organism such as *S*. *pyogenes* [118].

**Table 6:** Identity of putative phages and phage elements detected on the *S*. *pyogenes* genomes.

Sample Name	Phage Name	Position	No. of CDS	Size (Kbp)	GC %
**SP1**	P9 Phage*	543481–587686	58	44.2	39.5%
	ф315.2*^Δ^	1235237–1280343	59	45.1	38.6%
	ф315.2*	1753586–1838804	104	85.2	37.8%
	ф315.2*^Δ^	1836641–1850391	22	13.7	39.2%
	Temperate phage фNIH1.1*^Δ^	1856910–1921492	85	64.5	39.0%
**SP2**	ф315.2*^Δ^	36431–86156	70	49.7	36.9%
**SP3**	Enterococcus phage EFC-1	826891–851153	24	24.2	38.9%
	Bacillus phage Grass	1229568–1251639	22	22	39.7%
	ф315.4*	1702210–1746332	58	44.1	39.4%
**SP4**	Enterococcus phage EFC-1	1051712–1083210	27	31.4	36.8%
	ф315.6*^Δ^	1157140–1191141	45	34	39.2%
	ф315.2*	1678710–1710884	35	32.1	35.7%
	ф315.4*^Δ^	1815357–1861474	56	46.1	39.3%
	Temperate phage фNIH1.1*^Δ^	1865571–1906405	36	40.8	39.7%
**SP5**	ф315.3*	814075–836688	32	22.6	37.6%
	ф315.3*	940132–963402	31	23.2	38.5%
	P9 Phage*^Δ^	1206221–1259975	65	53.7	39.6%
	ф315.2*	1658995–1687543	24	28.5	36.3%
**SP6**	Temperate phage фNIH1.1*	485708–527064	52	41.3	39.0%
	Bacillus phage G	1041449–1074731	19	33.2	39.5%
	Shigella phage SfIV	1723507–1733053	11	9.5	42.1%
**SP7**	ф315.6*^Δ^	1113085–1154775	56	41.6	38.5%
	ф315.3*	1539782–1557690	27	17.9	36.4%
	ф315.4*^Δ^	1596065–1609564	22	13.5	36.7%
	Bacillus phage BCJA1c	1773969–1809715	32	35.7	35.8%
	ф315.4*	1804621–1885835	105	81.2	38.7%
	Bacillus phage G	1894717–1910959	25	16.2	42.2%
**SP8**	Temperate phage фNIH1.1*	580207–619535	53	39.3	38.3%
	ф315.2*^Δ^	1683466–1730855	68	47.3	38.0%
	P9 Phage*	1880672–1891879	23	11.2	40.0%
**SP10**	ф315.3*^Δ^	1401342–1434194	47	32.8	37.8%

* indicates Streptococcal specific phages

^Δ^ indicates confirmed complete phage sequences.

### Phylogeny

**Fig 3:**
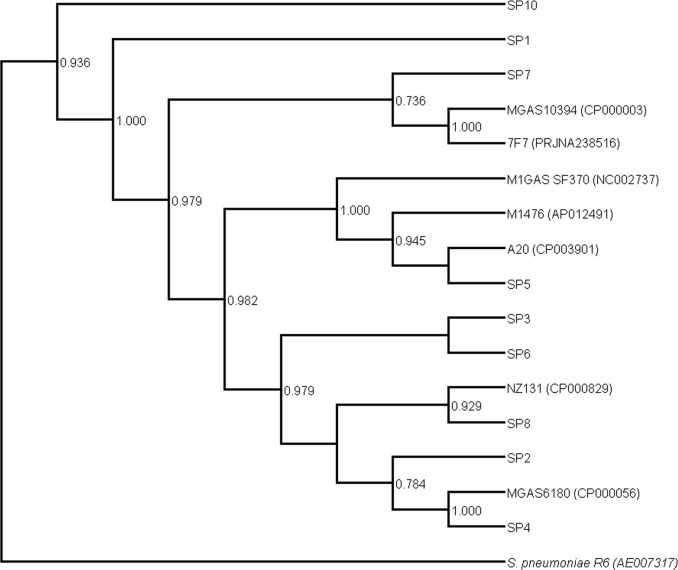
Phylogenetic tree of SP isolates and corresponding reference strains. The tree was constructed using 40 conserved coding marker genes of nine isolate and eight reference strain genomes. Three major clusters can be observed with bootstrap values on the nodes. The *S*. *pneumoniae* R6 strain is used as an outgroup for a more robust visualization.

**Fig 4:**
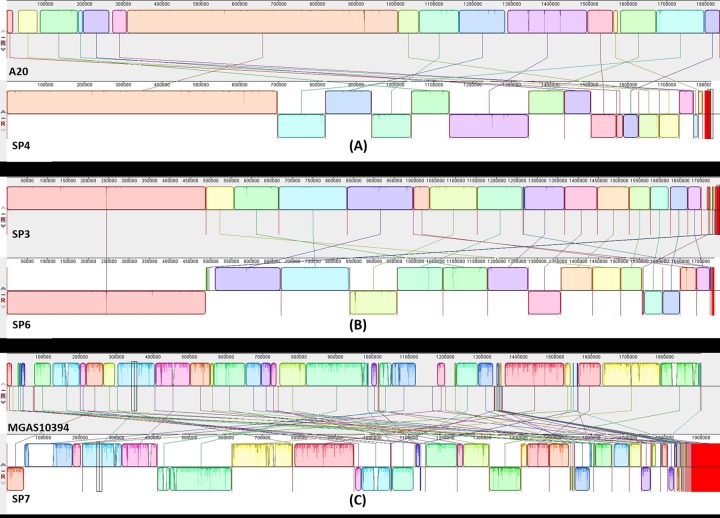
Genome comparison. Pairwise alignment of genomes from sub branches SP4, SP6, and SP7 with A20 (CP003901), SP3, and MGAS10394 (CP000003) respectively using the Mauve progressive alignment [25]. The colored blocks indicate homologous corresponding regions between the genomes that are internally free of rearrangement. Seismic lines inside blocks indicate the degree of similarity between alignments, while the red blocks indicate non-aligned sequences.

Coupling WGS with phylogenetic analysis has been shown to yield a high discriminatory power when dealing with closely related isolates, and has allowed for more robust epidemiological analyses [119, 120]. Here phylogenetic analysis was performed based on 40 gene families, revealing three major clusters [25] (Fig 3). As reported by Wu et al., the gene families used for the analysis are universally present across bacterial taxa, have a low copy number variation across taxa, and can be used to produce robust phylogenetic trees reflecting as much as possible the evolution of the species from which the genes have originated [121]. The genomic plasticity of *S*. *pyogenes* is manifested in the first phylogenetic branch through the clustering of SP7 with clonally and serotypically different reference strains (Table 1, Figs 3 and 4C). Sequence data of the MGAS10394 genome has revealed the presence of eight prophage-like elements [122], a fact in agreement with our findings of six phage-related elements at least on the SP7 genome (Table 6). Additionally, SP7 was one of the few genomes in the studied isolates to have the SpeA superantigen (Table 4), another characteristic of MGAS10394 [122]. Moreover, MGAS10394 was originally isolated from the throat of a patient with pharyngitis, and determined to be macrolide resistant [123], sharing both with SP7 isolate (Table 5).

In the second cluster, SP5 was grouped with the reference strains A20, M1476 and M1GAS SF370 indicating that these genomes are closely related given the fact that they all belong to the same clonal complex and *emm* type (*emm1*). In this context, it is noteworthy that SP5 was clinically isolated from a skin infection, and that the covRS system and the cysteine protease B superantigen were detected in its genome (Table 4). These findings are in harmony with the original study characterizing the A20 strain [124], again asserting a high similarity between the two genomes. Pairwise alignment of the two genomes also showed a high degree of homology with large parts of the of the two genome being preserved and homologous (Fig 4A).

In the third cluster, SP2, SP3, SP4, SP6 and SP8 were grouped closely with NZ131 and MGAS6180 indicating a high similarity and also reflective of the preliminary BLAST results. Within this branch, three additional subgroups can be distinguished. Being both clonal and of the same *emm* type (*emm89*/ST-101), SP3 and SP6 grouped very closely together and aligned with high sequence similarity (Figs 3 and 4B). Looking more closely at their genomes (Tables 4, 5 and 6), a very high similarity between genomic features can be seen, this sheds light on their close phylogenetic proximity. SP8 clustered with NZ131 whereas SP2 and SP4 grouped with the MGAS6180 strain with SP4 being much closer to the reference. Both SP4 and MGAS6180 are of the same *emm* and sequence types (*emm1* and ST-52) (Table 1). Interestingly, MGAS6180 is linked to puerperal sepsis with its virulence and pathogenesis attributed to of foreign gene uptake [94]. SP1 and SP10 did not group closely with any other isolate or reference strain (Fig 3), indicating considerable phylogenetic differences from their counterparts. Despite being associated with pharyngitis and skin infections, the distinct clustering of our isolates with different invasive strains, indicated potential invasiveness.

A recent study by Sanderson-Smith et al. [125], described a novel systematic and functional classification of *S*. *pyogenes* isolates into based on DNA sequencing of the full *emm* gene, and typed over 1000 GAS isolates in phylogenetic *emm*-clusters. Our phylogenetic analysis is in general agreement with the findings of this novel typing method; SP7 along with reference strains MGAS10394 and 7F7, clustered together and belonged to the D4 *emm*-cluster, SP3, 4, 6 grouped with MGAS6180 strain in the E4 *emm*-cluster, while SP5 clustered with the M1 serotype group of references M1GAS SF370, MGA1467, A20, collectively part of the A-C3 *emm*-cluster. SP1, phylogenetically clustering distantly from the rest, was also grouped in the A-C4 cluster *emm*-cluster phylogenetically further away from the previously mentioned clusters [125]. These findings further confirm that our isolates do not belong to a single phylogenetic cluster, but on the contrary, capture the range of serotypes that are implicated in throat and skin infections and that are present in the Lebanese community.

## Conclusion

Despite modern advancements in medicine, the disease burden of *S*. *pyogenes* remains very real especially in less developed countries. Its plethora of virulence factors and superantigens and its resistance to antimicrobial agents can quickly turn superficial infections into life threatening ones. The heterogeneity of stress response elements present on the streptococcal genome allows the organism to dynamically resist harsh conditions both inside and outside the host. The additional multiple gene regulation systems and their strict control over virulence factors in response to environmental conditions outline infections as being a highly transitional event closely dependent on external host stimuli. The high genomic plasticity characteristic of the species adds to the complexity of the identification process.

This study generated the draft genomes sequences of nine *S*. *pyogenes* clinical isolates and investigated their genomic content, invasive potential, antibiotic resistance, and examined their epidemiological origin through comparative phylogenetic methods. Regulation, signaling, and stress response elements were successfully identified in all of the isolates. Their overall virulence related elements being directly associated with throat and skin infections, without excluding their potential invasiveness. The hypervariable FCT region was found to be of Type-3 in all of the isolates excluding SP8, which exhibited an FCT-1 organization. The isolates showed large variation in the M protein family as well as in the FCT regulon. Seven distinct superantigen profiles were detected, showing common virulence traits between our isolates and those associated with pharyngitis and skin infections. Additionally, macrolide, fluoroquinolone, and rare resistance to tetracyclines were documented. Bacteriophages were detected in all of the isolates conferring superantigens as well as genes generally associated with invasive streptococcal diseases and resistance, a fact further confirmed by the phylogenetic study that linked our isolates to worldwide invasive-type clones. The phylogenetic study, in addition to the virulence profiles, showed a clear association between genome, site of isolation, and disease type. Moreover, we concluded that these strains did not form a single phylogenetic cluster, but instead captured the genomic diversity found in the reference strains.

In all, the detailed genome analysis will undoubtedly provide new insights on *S*. *pyogenes*. The expected merits of this study are not merely theoretical, as GAS remains an important cause of diseases in Lebanon and in the world, studying its functional genomics could help in better understanding the molecular mechanisms and epidemiology of its pathogenesis. Our findings add value to epidemiological studies of *S*. *pyogenes*, and provide the first such study for Lebanese isolates.
